# Saskatchewan Health Authority Library’s service innovation during the COVID-19 pandemic and receipt of the 2022 CHLA/ABSC Flower Award

**DOI:** 10.29173/jchla29762

**Published:** 2024-08-01

**Authors:** Courtney Ellsworth

**Affiliations:** Clinical Librarian, Saskatchewan Health Authority, Victoria Hospital, Prince Albert, SK

## Introduction

In the spring of 2020, COVID-19 was spreading globally and causing a rising tide of uncertainty amongst the public and healthcare personnel. Health organizations across the world had to react and make decisions that affected the safety and everyday life of their populations. The province of Saskatchewan struggled with advising healthcare professionals and the public on staying safe with a scarcity of evidence. Saskatchewan leaders urgently required scientific evidence related to COVID-19 to support decision-making in an attempt to control or minimize the spread of a potentially deadly disease.

The Saskatchewan Health Authority (SHA), formed in late 2017 from 12 separate health regions, provides integrated health services across the province. Many programs within the SHA employ evidence-based principles for patient care and to inform practice, which relies, in part, on using the most current data to make the best decisions in healthcare [1]. In response to the urgent demand and necessity of gathering new information to enact new policies and procedures during the early months of the COVID pandemic, an inter-institutional team was created to support decision making. This team consisted of the Saskatchewan Health Authority Library staff, SHA researchers and clinical experts, the University of Saskatchewan (U of S) clinical faculty, researchers, their library staff, and summer students, the Health Quality Council, and the Ministry of Health. This column describes the work the SHA Library performed to support these efforts and how the Library evolved its processes to function during the pandemic, culminating in earning the 2022 Flower Award for Innovation and Quality Improvement from the Canadian Health Libraries Association/ Association des bibliothèques de la santé du Canada (CHLA/ABSC).

### Emergency Operations Center & COVID-19 Evidence Support Team

The SHA created the Emergency Operations Center (EOC), comprised of physicians, VPs of the SHA, and other clinicians of the SHA, to manage its response to COVID-19. The COVID-19 Evidence Support Team (CEST) was established to clarify questions, effectively search the literature, and evaluate the evidence to create rapid reviews (RRs). CEST was formed through institutional partnerships, breaking down silos to work together. The library was invited to the meetings nine days after CEST was formed and quickly provided the evidence search support, and the technical and documentary backbone for the project. The library received COVID-19 information requests from the EOC via the project leader, Dr. Gary Groot, Professor, Department of Community Health and Epidemiology and Medical Director of Clinical Quality Improvement for the SHA. Search topics ranged from personal protective equipment, antibodies, epidemiology, vaccination, reinfection, and vulnerable populations, and helped inform provincial decision making. Librarians had to stay abreast of COVID-19 news, in the scientific field and public opinion, as misinformation and speculation created issues in the healthcare sphere that could predict and inform our workload. Notable searches include the potential dangers of infrared thermometers [2] because of concerns from the public that stemmed from misinformation on the internet. The library also handled searches on divisive topics such as re-opening schools [3], mask exemptions [4], and vaccine requirements [5]. Requests from SHA employees were funneled through the EOC to review their relevance to current questions and to identify their urgency. Once the need for a rapid review on a question was determined, the review process was initiated by librarians coordinating a reference interview.

### Searching & document creation

The majority of the library’s regular search service perform standard level three searches, wherein a complex search query is formulated and multiple information resources are utilized. For the COVID work supporting the EOC, a more rigorous process was required. The scarcity of quality information at the time, tight deadlines, and increasing amount of misinformation were daunting issues. Initially, requests for information would contain selected keywords with very little context, and impossible timeframes. The library had to establish a required reference interview in the process to refine the expectations of the search. The reference interview included the two assigned Librarians, the requestor, the reviewer, and possibly other team members. The interview outlined the scope of the request to confirm the concepts were understood to ensure relevant studies were captured in the search. At the beginning of the pandemic, timelines for the search results and reviews were extremely tight, sometimes within a few hours to a few days. While the urgency for evidence continued in the first few months of the pandemic, the requesting teams realized that timelines of 4 or 8 hours were impossible to achieve and even harder to sustain. To reduce the time to produce the evidence search reports (ESRs), Librarians divided the work with one Librarian focusing on searching bibliographic databases such as MEDLINE, Embase, and CINAHL, and one librarian conducting the grey literature search. Many publishers had opened their journal literature during the pandemic but access to Embase was purchased to complement the existing database collection.

To guide and standardize searching, the library created an internal document with grey literature sources specific to COVID-19, including the World Health Organization Coronavirus Disease (COVID-19) Pandemic site [6], the CDC COVID-19 site [7], COVID-19 Evidence Alerts from McMaster PLUS [8], NCCMT COVID-19 Rapid Evidence Reviews [9], L-OVE COVID-19 [10], and LTC Covid [11], among many others. Sources were constantly sought, evaluated, added, and removed over time. This internal document of grey literature was invaluable to the project, as it saved time and created consistency across teams. It also included sources with preprints (unpublished manuscripts of health science literature) such as medRxiv [12]. Although unpublished and therefore not yet peer-reviewed, these manuscripts contained vital information related to COVID-19. Since new insights into COVID-19 could not wait for published studies, the unpublished materials were immensely valuable.

The ESRs produced by the librarians contained the following: search question, the requestor name, recorded search strategies for bibliographic databases and grey literature, search locations (organizations’ websites, databases, news websites, etc.), organized grey literature links, and a numbered list of abstracts of journal articles. Once ready, the librarians then submitted the ESR to a CEST research scientist, who synthesized and graded the evidence to create a RR. The RRs were circulated to the project lead and requesting team who shared the evidence with senior leaders in the SHA.

### The repository with dashboard

Initially, the library’s role was focused on performing the literature searches. It became apparent that more structure was required to control the questions asked to avoid duplicate or similar searches and to monitor the volume of work coming in from which groups (EOC, LTC, public health, critical care, etc.). New document templates and a way to share the information with the CEST team were also required. Using its connections with the Research Department within the SHA, the library collaborated with a research scientist to create a template for the RRs that they would also be using, and the librarians created a template for the ESRs based on the standard evidence search report already utilized in the library.

The CEST team needed a dashboard to track the subject areas of the questions, the number of questions, and the status of the work. To avoid duplicate data entry and create automated processes, the library used tools already available and minimized the need for IT support. These tools included RedCap (a research survey tool), LibGuides, DB/Textworks (a searchable database management system), and AnDI (a web-based interface that interacted automatically with the database). The library team used Trello, a web-based application that uses cards to manage the workflow of projects, to track their work.

Once the reference interview was conducted, the lead librarian would complete a RedCap form (Online Supplement, Appendix - COVID-19 evidence review request) that included fields regarding the urgency, the subject area, the affected population, and requester. The completed RedCap form was automatically imported into a DB/Textworks database (database 1). A report was then created to email the question details to the requesting team to confirm receipt. The System Library Technician generated a notification report from the database and the Technician emailed the designated library team. The record was also exported to populate the dashboard, which resides on the library's COVID-19 Repository page [13] created specifically for the project.

Supplement, Appendix

When the ESR was completed, a record was created in a database (database 2) linked to database 1. Database 2 housed the ESRs and the RRs. Information was updated about the status in the databases (such as request submitted, evidence search in progress, completed, etc.), which automatically populated the dashboard. When searching the library's COVID-19 database [14] on the repository, fields from both databases were used in the record displays. The linked databases reduced duplicating data entry.

Andornot Consulting provided valuable technical support. They provided licenses to DB/Textworks and provided the web-based interface, AnDI. Their knowledge and expertise made automating steps of the work possible by writing the scripts to pull records from RedCap or the databases to create the dashboard and other reports. The SHA IT department was not able to support the project as they were involved in other COVID work.

AnDI automatically published the ESR and RR documents to the website, made them discoverable by using the search feature, and created the permalinks for easy sharing and the citation fields for proper referencing. Andornot Consulting also facilitated the creation of a report format that allows users to export the RRs from the database as an RIS file. The repository was created on April 15 2020 and initially it was only available to the CEST team. However, it was made available to the public in August 2020 to facilitate information sharing between organizations.

To capture statistics on the number of ESRs and RRs and the amount of time spent producing them, records were exported from the DB/Textworks database to an Excel pivot table. The CEST team shared the statistics with senior leaders to ensure the project continued to receive funding and personnel. The statistics were also incorporated into various research activities on the project.

### Day-to-day operations

During this time, the library’s regular services, such as standard evidence searching, reference questions, article retrieval, and teaching, never ceased functioning. Six of the seven physical locations were temporarily closed and the space was used by other departments. During the height of the pandemic, the library transitioned to remote work using employer-issued laptops, effectively redesigning primary service delivery. The change to virtual engagement was ultimately beneficial, as online delivery across the organization was enhanced and is still utilized. Clinical staff became comfortable with virtual meeting software, such as WebEx, and technology such as webcams, which made virtual reference interviews more common, especially with COVID-19 searches. Library staff continue to use WebEx to instruct and provide reference interviews with staff across the province with ease.

## Conclusion

The COVID-19 pandemic brought many challenges to the SHA Library, but also many opportunities to display adaptiveness and resilience by evolving services to meet the information needs of its users. CEST activity concluded on June 30, 2022 having produced a total of 246 ESRs and 185 RRs. The repository remains accessible from the library’s website. The library exemplified creativity and innovation by creating new databases with automated processes to support CEST and the EOC, thus earning them the Flower Award for Innovation and Quality Improvement from the Canadian Health Libraries Association (CHLA/ABSC). The SHA Library now has the procedures and resources to respond to the next global event that will require synthesized information for policy-level decision making. All health libraries had to work through this pandemic with increased demand and stress levels. Hopefully, the value of health libraries and their services as a whole has been noted in light of this incredibly difficult time.
