# Supplementary material for: Saskatchewan Health Authority Library’s service innovation during the COVID-19 pandemic and receipt of the 2022 CHLA/ABSC Flower Award
Source: J Can Health Libr Assoc. 2024 Aug 1;45(2):98–102. doi: 10.29173/jchla29762 (PMC11485156; doi:10.29173/jchla29762)
Supplement: Supplementary file 1 [file JCHLA-45-098-s001.pdf]

Appendix -
COVID-19 Evidence Review Request

Research Teams will be sent a confirmation of the request which will include the request's Unique Identifier. The Unique Identifier must be used on the Evidence Search Report and Rapid Review.

Research Question

____
(Example: What laboratory tests should be conducted in the general population?)

Priority level - refers to completion of evidence search & rapid review

- ◯ Level 1 2-3 days
- ◯ Level 2 One week (7 days)
- ◯ Level 3 Two weeks (14 days)
- ◯ Level 4 Three weeks (21 days)
- ◯ Level 5 Four weeks+ (28 days+)

Review to be used for

- ☐ Policy development
- ☐ Public information
- ☐ Clinical Standards development
- ☐ Other

Other purpose for review

____

Context

____
(Example: Consider symptomatic and/or asymptomatic individuals; total population or sample; what tests (specify). Librarian will need to confirm the context with the team.)

Research Team

- ◯ Long Term Care
- ◯ Epidemiology & Modelling
- ◯ Public Health
- ◯ Therapeutics
- ◯ Infectious Disease
- ◯ Policy
- ◯ Clinical/Acute Care
- ◯ EOC
- ◯ Personal Protective Equipment
- ◯ Laboratory
- ◯ Indigenous Health
- ◯ Research Team Pending
- ◯ Critical Care

Categories. Select up to 2.

- ☐ Administration
- ☐ Clinical Management
- ☐ Clinical Presentation
- ☐ Diagnostics
- ☐ Epidemiology
- ☐ Healthcare Services
- ☐ Infection Prevention and Control

Population - Check all that apply	☐ All<br>☐ Neonates<br>☐ Infants<br>☐ All Pediatrics<br>☐ All adults<br>☐ Aged (80+)<br>☐ Homeless<br>☐ Mental Health patients<br>☐ Indigenous Peoples<br>☐ Other
Other Population	__________________________________
Clinical Setting	☐ Ambulatory<br>☐ Cardiac unit<br>☐ Community<br>☐ Dialysis unit<br>☐ Emergency<br>☐ EMS<br>☐ ICU<br>☐ Long Term Care<br>☐ Medicine Unit<br>☐ NICU<br>☐ Oncology<br>☐ Primary care<br>☐ Public Health<br>☐ Other
Other Clinical Setting	__________________________________
Requester First Name	____________________________
Requester Last Name	____________________________
Organization	____________________________
Email	____________________________
Phone	____________________________
Cell Phone	____________________________
Best method for quick contact	◯ Email<br>◯ Phone<br>◯ Cell Phone
Date the question was originally asked on.	____________________________

Additional Information (i.e. similar study, document or evidence). Librarians, please provide your names here. If this question is on hold, please indicate.	________________________________
Status of review. Please select one.	◯ 1. Search in progress<br>◯ 2. Review in progress<br>◯ 3. Completed
